# Insights into the Mechanisms Involved in Protective Effects of VEGF-B in Dopaminergic Neurons

**DOI:** 10.1155/2017/4263795

**Published:** 2017-04-03

**Authors:** Beatrice Caballero, Scott J. Sherman, Torsten Falk

**Affiliations:** ^1^Department of Neurology, College of Medicine, University of Arizona, Tucson, AZ 85724, USA; ^2^Department of Cellular and Molecular Medicine, College of Medicine, University of Arizona, Tucson, AZ 85724, USA; ^3^Department of Pharmacology, College of Medicine, University of Arizona, Tucson, AZ 85724, USA

## Abstract

Vascular endothelial growth factor-B (VEGF-B), when initially discovered, was thought to be an angiogenic factor, due to its intimate sequence homology and receptor binding similarity to the prototype angiogenic factor, vascular endothelial growth factor-A (VEGF-A). Studies demonstrated that VEGF-B, unlike VEGF-A, did not play a significant role in angiogenesis or vascular permeability and has become an active area of interest because of its role as a survival factor in pathological processes in a multitude of systems, including the brain. By characterization of important downstream targets of VEGF-B that regulate different cellular processes in the nervous system and cardiovascular system, it may be possible to develop more effective clinical interventions in diseases such as Parkinson's disease (PD), Amyotrophic Lateral Sclerosis (ALS), and ischemic heart disease, which all share mitochondrial dysfunction as part of the disease. Here we summarize what is currently known about the mechanism of action of VEGF-B in pathological processes. We explore its potential as a homeostatic protective factor that improves mitochondrial function in the setting of cardiovascular and neurological disease, with a specific focus on dopaminergic neurons in Parkinson's disease.

## 1. Introduction 

Parkinson's disease (PD) is a progressive neurodegenerative disease affecting approximately 1.5 million people in the United States. This disease involves the loss of neurons of the substantia nigra pars compacta (SNpc), as well as the loss of dopaminergic nerve terminals in its target area, the striatum. Lewy bodies, abnormal aggregates of protein, are the pathological hallmark of PD. The diminishing number of dopaminergic neurons ultimately leads to the depletion of dopamine content in the striatum [1] and results in a variety of motor and nonmotor deficits [2]. There are several theories behind the pathogenesis of PD but all hold in common a central idea of mitochondrial dysfunction among both sporadic and familial forms of the disease. Mitochondrial dysfunction may be the result of bioenergetics defects, mitochondrial DNA mutations, alteration in mitochondrial dynamics, and presence of mutated proteins associated with mitochondria [3]. Defects in mitochondrial respiration are involved in human PD, as demonstrated in a study reporting accidental infusions of the toxin 1-methyl-4-phenyl-1,2,3, 6-tetrahydropyridine (MPTP), which selectively inhibits a component of the electron transport chain, mitochondrial complex 1 [4, 5]. This toxin is selectively taken up by dopaminergic neurons, leading to cell loss in the SNpc and a Parkinsonian state. Other toxins, such as the pesticide rotenone, also inhibit complex 1, inducing dopaminergic degeneration in humans and rodents, supporting the idea that mitochondrial dysfunction plays a central role in the pathogenesis of PD [6]. The “multiple hit” hypothesis of the pathogenesis of PD suggests that multiple insults have to come together to cause PD [7]. Calcium-induced toxicity may be one of the “hits” contributing to the selective vulnerability of dopaminergic neurons in PD. It has been observed that engagement of L-type calcium channels during autonomous pacemaking increases the sensitivity of SNpc dopaminergic neurons to mitochondrial toxins in animal models of PD. Human epidemiological data also supports a linkage between L-type calcium channels and the risk of developing PD [8]. There are also implications of genetic mutations in mitochondrial dysfunction. DJ-1, encoded by the* PARK7* gene, protects neurons against oxidative stress and cell death. Deletion of DJ-1 exacerbated elevated mitochondrial oxidant stress due to calcium entry, demonstrating that neurodegenerative changes in PD may be driven by metabolic stress created by calcium entry, particularly in the face of genetic factors that compromise defense mechanisms [8]. The first gene to be linked to familial PD was* SNCA*, which encodes the protein *α*-synuclein, and since *α*-synuclein is also a main contributor to Lewy bodies in idiopathic PD it is of special importance for PD. Both monomeric and oligomeric forms of *α*-synuclein have differential effects on mitochondria, including increasing calcium, inhibitory effects on complex I activity, and inducing reactive oxygen species (ROS), as reviewed in [9]. Another familial form of early-onset PD can be caused by mutations in PTEN-induced kinase 1 (PINK1), where PINK1 mutations or knockdown of PINK1 results in an increase of *α*-synuclein aggregates in cell-culture PD models, decrease in mitochondrial respiration, and a decrease in ATP synthesis [3]. Functionally, PINK1 is a kinase and phosphorylates parkin and ubiquitin to regulate mitophagy* (vide infra)*. And finally, the* Parkin* gene, the causative gene for an autosomal recessive form of PD, has been shown to be necessary for autophagy of mitochondria. The connection between PD and mitochondria suggests that malfunction in the mitochondrial quality control process can lead to an accumulation of defective mitochondria and cell death [10].

Properties maintaining mitochondrial homeostasis are collectively known as mitochondrial dynamics and these processes, such as mitochondrial fission, fusion, and transport, interact to maintain the electron transport chain function, prevent buildup of damaged proteins, control mitochondrial turnover, and regulate cell death processes. Defects in any of these processes may be involved in PD pathogenesis [11]. With regard to PD, a significant amount of evidence supports the role of mitochondrial dysregulation in the pathophysiology of this disease. The high vulnerability of dopaminergic neurons to metabolic dysfunction may be due to their elevated bioenergetic requirements and particular morphological characteristics of these neurons such as a high density of axonal mitochondria and complex axonal arborizations [12].

Current treatments such as l-dihydroxyphenylalanine (L-DOPA) and deep brain stimulation, while effective, do not address the underlying issue of neurodegeneration involved in PD and serve to provide only symptomatic relief. Because these methods do not address the core cause of PD nor halt disease progression, there is need for improved therapeutic options aimed at identifying the cause of dopaminergic cell loss.

Similarities may exist among signaling pathways during pathological states involving the nervous system and cardiovascular system, and reviewing the role of VEGF-B can provide new perspectives for medically important diseases. The role of VEGF-B remains ambiguous compared to VEGF-A, which is the archetypal angiogenic factor and the most extensively studied among the VEGF family [13]. VEGF-B has the potential to protect both cardiomyocytes from ischemic injury and neurons from degeneration. It is important to identify the similarities and differences between VEGF-B actions in these processes in order to determine areas of further research.

## 2. Vascular Endothelial Grown Factor Family

Vascular endothelial growth factor (VEGF) was first isolated in 1983 by Senger and colleagues and identified as vascular permeability factor (VPF) due to its ability to stimulate vascular permeability [14]. However, in 1989 Napoleone Ferrara and colleagues purified the same protein, which was distinguished for its role as a potent endothelial mitogen. It was then renamed “vascular endothelial growth factor” (VEGF). The protein purified by Ferrara and colleagues was the most biologically active isoform of the VEGF family, VEGF-A [15]. Although VEGF-A is the most studied isoform, there are 4 other identified members that make up the VEGF family, VEGF-B, VEGF-C, VEGF-D, and PlGF (placental growth factor) [16]. The VEGF family is known to bind to three receptor tyrosine kinases, known as vascular endothelial growth factor receptor-1, -2, and -3 (VEGFR-1–3; sometimes also referred to as flt-1, flk-1, and flt-4), activating several signaling cascades which regulate a multitude of cellular functions [17]. VEGFRs have an approximately 750-amino-acid residue extracellular domain organized into seven immunoglobulin-like folds as well as a single transmembrane region and an intracellular tyrosine kinase domain interrupted by a kinase-insert domain [16]. The VEGF ligands bind to these three main receptors with differing specificities. VEGFs are dimeric glycoproteins of approximately 40 kDa that bind the RTKs and cross-link them as dimers. As a result, the RTK's cytoplasmic tyrosine kinase domains autophosphorylate, which then triggers an intracellular signaling cascade that transduces the signal initiated by VEGF-binding into the cell [17]. This paper will focus on VEGFR-1 and VEGFR-2 regulators and their interactions with VEGF ligands. Both VEGFR-1 and VEGFR-2 are high affinity receptors for VEGF-A, while VEGF-B only interacts with VEGFR-1 [17]. Most functional VEGF-A signaling is mediated via VEGFR-2 and upon VEGF-A-induced stimulation of this receptor, there are major proangiogenic signals generated [18], while protective mechanisms in both the brain and the heart can involve signaling mediated through VEGFR-1 receptors [19, 20]. These different interactions determine downstream effects and allow for the comparison between VEGF-A and VEGF-B functions in various processes. Though VEGF-A is the most extensively studied of the VEGF family, multiple studies determined the protective role of VEGF-B in diseases such as ischemic heart disease and neurodegenerative diseases.

## 3. Why VEGF-B?

VEGF family members are key regulators of vascular biology, modulating angiogenesis, vasculogenesis, and maintaining vasculature during embryogenesis and in adults. VEGF-A has been considerably studied on the account of its ability to promote angiogenic effects via one of its receptors, VEGFR-2 [16]. VEGF-A can be induced when cells are subjected to hypoxic conditions and plays a central role in neovascularization, increasing energy substrates and oxygen. Hypoxia induces hypoxia inducible factor-1 expression, which binds to the enhancer sequence of the VEGF-A gene and mediates its physiological functions [21]. In contrast, VEGF-B does not contain a hypoxia response element in the promoter region and is not transcriptionally induced by hypoxia. However, VEGF-B is induced by ischemia in the brain but another unidentified mechanism must be involved in the stimulation of VEGF-B. VEGF-A is one of the strongest inducers of vascular permeability [22] and has also been shown to have neuroprotective effects in both* in vitro* and* in vivo* PD models [23]. For example, a study conducted by Yasuhara et al. suggested that VEGF-A effectively protects against 6-hydroxydopamine-induced cell death within cultures of embryonic rat mesencephalic dopaminergic neurons. Likewise, the continuous injection of VEGF-A, via encapsulated VEGF-A secreting cells (engineered baby hamster kidney cells), into the right striatum of adult rats resulted in a neuroprotective effect. There was a rescue of dopaminergic neurons demonstrated by an increase in tyrosine hydroxylase- (TH-) positive neurons in the striatum as well as a functional improvement demonstrated by a significant decrease in amphetamine-induced rotational behavior [23]. Another study supported the protective effect of VEGF-A, concluding that the intrastriatal delivery of VEGF-expressing adeno-associated virus demonstrated favorable effects on dopaminergic neurons in a rat Parkinson's disease model [24]. A direct neurotrophic effect on dopaminergic neurons by VEGF-A was suggested given that VEGF-A increased both tyrosine hydroxylase-immunoreactive cell survival and neurite outgrowth* in vitro* [25]. Similar protective effects were demonstrated in a study by Silverman et al. where VEGF-A promoted survival particularly of dopaminergic neurons in rat midbrain explant cultures [26].

While neuroprotective effects of VEGF-A have been identified, administering VEGF-A at high levels may lead to unwanted side effects such as an increase in vessel density [27] and cerebral edema [28], due to its angiogenic activity. VEGF-A has also been recently indicated in contributing to the development of L-DOPA-induced dyskinesia (LID), a dose limiting side effect of the gold standard treatment for PD, by increasing microvascular density [29]. Given these side effects, other studies investigated another VEGF isoform, VEGF-B, which may have a similar action in certain diseases but without these unwanted effects, due to the lack of angiogenic function. In contrast to VEGF-A, VEGF-B does not contain a hypoxia response element in the promoter region and is not transcriptionally induced by hypoxia. However, VEGF-B is induced by ischemia in the brain. VEGF-B upregulation in other pathological processes such as cortical cord injury in rats [30], motor neuron degeneration [31], and a cell-culture model of PD has also been shown [32]. In both ALS and peripheral neuron disease, VEGF-B mediated protective effects against neuronal degeneration [31, 33, 34]. VEGF-B evidently plays a role in various pathological processes, some of which will be discussed further in this review. The mechanisms of action, however, remain unclear and not well established. Nonetheless, VEGF-B protective function is an active area of research that merits investigation given its differences in safety profile.

## 4. Tissue Expression of VEGF-B and VEGFR-1

In order to elaborate on the action of VEGF-B and consider VEGFR-1 as part of its signaling pathway in certain diseases, it is important to determine expression of both VEGF-B and VEGR-1 in areas of interest, such as the heart and brain.* VEGF-B* expression in mouse tissues and human tissues was determined by Northern blot analysis. In mouse tissues,* VEGF-B* transcript expression was most abundant in heart, brain, lung, skeletal muscle, and kidney. In human tissues,* VEGF-B* transcript expression was most abundant in heart, skeletal muscle, pancreas, and prostate [35]. In a study by Muhl et al. the expression of both* VEGF-B* and* VEGFR-1* was explored in the murine heart, lung, and kidney. In all organs, there was a cell-specific pattern with VEGFR-1 expression restricted to endothelial cells and VEGF-B expression was found in cardiomyocytes, pulmonary myocardium of the lung, and renal epithelial cells of the kidney [36]. During ischemic stroke, VEGF-B expression was increased in neurons and inflammatory (macrophage/microglial) cells of the ischemic border zone after cerebral artery occlusion in a rat model [37]. In order for VEGF-B to affect motor neurons, VEGF-B must be expressed in the ventral horn of the spinal cord. Immunohistochemistry revealed VEGF-B expression in large neurons in the ventral horn, which were considered to be motor neurons given their size and location; expression was also shown in dorsal root ganglion neurons and in blood vessels [31]. VEGF-B was not detectable in quiescent astroglia in SOD1 mice, but its expression became prominent in activated astrocytes thought to maintain survival of motor neurons after injury [31]. Another example of VEGF-B expression in pathological states comes from a study by Boer et al. in which expression of VEGF-B and its receptor VEGFR-1 was demonstrated in resected human brain tissue from patients undergoing epilepsy surgery for focal cortical dysplasia type IIB [38]. Histologically normal cortex displayed only weak VEGF-B immunoreactivity, but an increased expression in the dysplastic cortex. Expression was shown in pyramidal neurons, in endothelial cells, and in CD68+ macrophages, while glial cells did not show VEGF-B staining. VEGF-B and VEGFR-1 are also expressed in neuromelanin-containing neurons in the SNpc of both healthy controls and PD patients; microglial and vascular immunoreactivity was also present in the SNpc [19, 39]. There were no overt differences between control subjects and PD patients in this small cohort.

## 5. Link between Cardiovascular System and Nervous System

**Figure 1:**
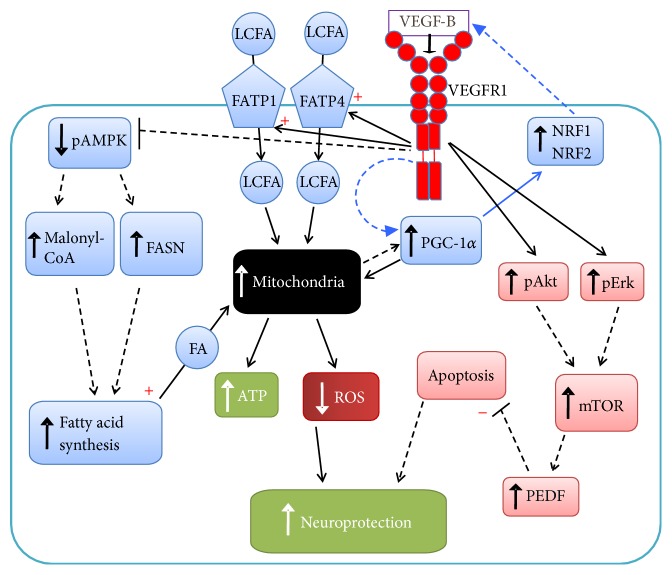
Proposed mechanisms of VEGF-B's protective effects in dopaminergic neurons. Black solid lines indicate pathways previously shown for DA cells. Black dashed lines indicate hypothesized pathways, based on other cell types. Blue lines indicate a possible feedback loop between VEGF-B and PGC-1*α*. Arrowheads and + signs: activation, − signs: inhibition. LCFA: long chain fatty acids; FA: fatty acids. In neuron survival, VEGF-B via VEGFR-1 upregulates pAkt and to a lesser extent pErk. Given data seen in cardiac tissue and sensory neurons, we propose that mTOR is increased downstream and generates antiapoptotic effects via upregulation of PEDF. Previous data also shows that VEGF-B mediated upregulation of fatty acid transporters FATP1 and FATP4 via VEGFR-1, leading to LCFA uptake and increasing mitochondrial function. This could generate more ATP and decreases ROS, promoting neuronal protection. Additionally, given VEGF-B action on pAMPK in cardiac cell protection, reduced AMPK phosphorylation may be involved in neuroprotection and serves as another way of improving mitochondrial function during neuron injury. The nuclear respiratory factors, NRF1 and NRF2, are downstream of PGC-1*α* in neuroprotection, and given findings in skeletal muscle, VEGF-B may be regulated by PGC-1*α*, via NRF1 and NRF2. Applying data from prior work in cardiac cells, we also suggest that VEGF-B may increase PGC-1*α* expression, providing an autocrine feedback loop in which either VEGF-B is directly upregulating PGC-1*α* followed by increased mitochondrial activity or VEGF-B could be increasing mitochondrial activity that is then feeding back to increase PGC-1*α* in order to maintain increased ATP production.

Cells in both the heart and the brain are energy dependent cells, requiring tight control of mitochondria and subsequent cell functions. Perhaps if nonfunctional or damaged mitochondria begin to accumulate in neurons or cardiomyocytes, the energy required to maintain proper cell function is insufficient to promote cell survival in the presence of a pathological process. VEGF-B has been found to have protective capabilities for cells during injury in various contexts [33, 40, 41], and given its role in pathology involving both systems, future studies should continue elucidating the molecular links associated with cardiovascular and neurological diseases. Since mitochondrial dysfunction is central in PD, for example, VEGF-B mediated increases in fatty acid transport could help during disturbances in oxidative stress that cause mitochondrial dysfunction by providing an energy source for viable cells [42]. Generally, VEGFR-1, VEGF-B's receptor, activates downstream pathways used to aid with protective processes during cellular stress [16]. These pathways include regulation of the Akt pathway and Fatty Acid Transport Proteins (FATP), downstream factors that have been involved in VEGF-B protective function (Figure 1). This shared feature can potentially serve as a link between both systems that can provide a learning opportunity about new areas of research and more insight into the development of new therapeutic agents that target specific pathways in certain disease processes.

## 6. Neuroprotective Role of VEGF-B in Parkinson's Disease

Growth factors have shown preclinical promise for treatment of PD, as reviewed in [43, 44], but have not yet been translated into the clinic. Combinations of more than one neurotrophic factor might be ultimately necessary for clinical efficacy [45]. To help with identifying additional protective factor candidates with distinct mechanisms of action, a study by Falk et al. used a rat midbrain culture model to identify genes that are changed after the addition of a neurotoxin, rotenone. Rotenone is a naturally occurring toxin and a commonly used pesticide that reproduces the neuropathological and behavioral features of PD in rats and is a well-characterized model for this disease [46]. In this study a gene chip array analysis demonstrated several genes were upregulated after treatment with rotenone and transcriptional activation of VEGF-B was evident. To verify these results, a semiquantitative western analysis was conducted, which demonstrated increased protein expression of VEGF-B, proving VEGF-B to be an inducible factor in a model of neurodegeneration [32]. To investigate the role of VEGF-B specifically, this same study utilized a rat midbrain* in vitro* PD model. In one series of experiments, there was a concentration dependent protective effect of VEGF-B on primary neurons in culture, with a mean effect of 30% increase in TH-positive cell number when compared to untreated cells. This reflected VEGF-B's protective effect against the natural loss of dopaminergic neurons in culture. Another series of experiments in the same study involved testing additional exogenous VEGF-B in the midbrain culture model using a severe rotenone challenge. This rotenone challenge resulted in a significant reduction of TH-positive neurons per culture dish compared to untreated cells (control). One hour prior to rotenone challenge, VEGF-B was administered and a neuroprotective effect of VEGF-B rescuing cell loss was demonstrated [32]. Based on the results using an* in vitro* PD model, VEGF-B's neuroprotective effect was investigated in an* in vivo* PD model. This study by Falk et al. involved utilizing a mildly progressive unilateral 6-hydroxydopamine (OHDA) rat PD model with an intrastriatal injection of VEGF-B prior to the neurotoxic 6-OHDA treatment. In VEGF-B treated animals, there was an improvement in PD behavior, indicating a protective effect of VEGF-B [19, 47]. Further analysis demonstrated that VEGF-B partially protected dopaminergic fibers in the striatum and partially rescued dopaminergic neurons in the SNpc [19, 47]. To identify genes that are coexpressed with* VEGF-B* and establish links to known signaling pathways or metabolic networks, Hagberg et al. clustered microarray data, which showed a significant coexpression of* VEGF-B* with nuclear encoded mitochondrial genes involved in fatty acid metabolism [42]. To identify potential mechanisms of VEGF-B's neuroprotective action on dopaminergic neurons, Yue et al. used the human neuroblastoma cell line SH-SY5Y, which exhibits hallmark characteristics of dopaminergic cells [48] and is a common cellular PD model. Certain pathways were chosen as potential targets of VEGFR-1 based on evidence from other tissue types. Among these targets are fatty acid transport protein 1 (FATP1), fatty acid transport protein 4 (FATP4), and Akt.

Transport of long chain fatty acids (LCFA) across the plasma membrane is facilitated by FATPs, providing an important source of energy for most organisms. FATPs have been shown to be expressed in a variety of tissues [49], and of particular importance is the expression of the murine FATP1 and FATP4 in the brain [49]. This expression of FATP1 and FATP4 in the brain is fundamental, as this was the primary area of interest. Taking also into consideration the regulation FATP transcription by VEGF-B [42], FATP1 and FATP4 were of specific interest.

In addition to exploring FATP1 and FATP4, investigating pathways involved in regulating transcription of antiapoptotic genes was another objective. In a study by Li et al. Akt pathways were shown to play a role in VEGF-B's inhibitory effect on apoptosis using different cell lines [50]. Given these findings, the potential role of Akt and Erk 1/2 in VEGF-B's neuroprotective effect on dopaminergic cells was also investigated.

After investigating these pathways it was determined that the neuroprotective mechanisms of VEGF-B involve upregulation of FATP1 and FATP4 and activation of Akt and Erk1/2 signaling pathways. There was an evident upregulation of FATP1 and FATP4 with VEGF-B cotreatment after rotenone-induced stress [19], and the VEGF-B-induced increase of FATP1 and FATP4 mediated by VEGFR-1 may facilitate the translocation of long chain fatty acids across the plasma membrane, increasing mitochondrial function and allowing cells to recover from rotenone-induced mitochondrial damage by providing an additional energy source.

This is especially interesting since the mitochondria are thought to be at the center of both genetic and potential environmental causes for PD, and dopaminergic neurons in the SNpc are known to have a high mitochondrial oxidant stress, due to their high metabolic demands to maintain dopamine release [8, 51].

VEGF-B cotreatment significantly reversed rotenone-induced downregulation of total Akt and phospho-Akt protein levels, at both phosphorylation sites that are required together for maximal activation, and to a minimal degree, VEGF-B activated Erk 1/2. The increased Akt and Erk 1/2 signaling could be a parallel or additive process leading directly to activation of antiapoptotic cellular cascades, as has been shown in other tissues, including neuronal cells [50].

In summary, it was shown that the mechanisms of VEGF-B's neuroprotective action can involve VEGFR-1 mediated upregulation of FATP1 and FATP4 and activation of the Akt and Erk 1/2 signaling pathways [19] (Figure 1). By characterization of downstream targets of VEGF-B that are important in regulating cell processes in the brain, especially dopaminergic neurons, it may be possible to develop more effective clinical interventions to promote neuronal protection in PD and other neurodegenerative diseases involving mitochondrial dysfunction.

## 7. Neuroprotective Role of VEGF-B in Amyotrophic Lateral Sclerosis

Amyotrophic Lateral Sclerosis (ALS), a fatal and devastating adult-onset neurodegenerative disorder characterized by rapidly progressive degeneration of motor neurons in the spinal cord, brainstem, and primary motor cortex, is another disorder in which VEGF-A has been shown to play a major role. In one particular study, VEGF-A had promising results, with neuroprotection of motor neurons significantly improving neuronal survival in animal models [52]. However, as previously mentioned, the issues with VEGF-A's safety profile remain. The undesirable effects of VEGF-A on capillary permeability and the inflammatory response in the brain warrant studying another therapeutic pathway with a higher safety potential. In a study by Poesen et al. the role of VEGF-B and VEGFR-1 in motor degeneration in rodent models of ALS was investigated [31]. VEGF-B should be present in the ventral horn of the spinal cord, for it to affect motor neurons; therefore, expression was determined in WT mice and real time PCR analysis revealed VEGF-B was present in WT mice embryos and adult mice. Mice lacking VEGF-B developed a more severe form of motor neuron degeneration when intercrossed with mutant superoxide dismutase 1 (SOD1) mice, a model for ALS. And intracerebroventricular VEGF-B recombinant protein injection prolonged survival of SOD1 mice, without causing blood vessel growth or blood-brain barrier leakiness. VEGF-B was not detectable in quiescent glial cells in presymptomatic SOD1 mice, but its expression was progressively upregulated in activated glial cells in symptomatic SOD1 mice, indicating a possible stress-induced paracrine effect. Given VEGFR-1 is the receptor for VEGF-B; this study also confirmed expression of VEGFR-1 in mouse spinal cord by RT-PCR analysis of VEGFR-1 transcript levels. After isolating primary motor neurons from embryonic mice embryos, VEGF-B was added to determine if it protected motor neurons from cell death, which came from deprivation of growth supplements. After comparing the percentage of surviving motor neurons to the initial number of motor neurons, VEGF-B was shown to significantly increase survival in a dose dependent manner. To investigate how VEGF-B mediated its neuroprotective effects, Poesen et al. used mice expressing a tyrosine kinase-dead VEGFR-1, which cannot conduct the biological effect of VEGF-B. As predicted, they found that VEGF-B failed to protect embryonic VEGF-1R-TK^−/−^ motor neurons, indicating that VEGF-B exerts neuroprotective activity through VEGFR-1 mediated signaling [31]. The signaling cascade downstream of VEGFR-1, however, is not well understood.

The pathological mechanism in ALS is multifactorial and complex. Oxidative phosphorylation in mitochondria is the major source for reactive oxygen species and oxidative stress and mitochondrial damage are just one link that has been investigated in the pathogenesis of ALS [53]. Given the multiple functions of the mitochondria, damage to this organelle or alteration of its properties might confer susceptibility of motor neurons to stress and result in cell death. If mitochondria accumulate in the cell in a dysfunctional state, it is no longer able to supply ATP to the neuromuscular junction or to the cell body, eventually resulting in the degeneration of distal synapses and death of motor neurons [53]. Using information from VEGF-B action in PD, we could hypothesize the mechanism of neuroprotection in ALS as well. There is a possible role for VEGF-B in helping mitochondria with compromised function survive by mediating upregulation of fatty acid transport into cells to be utilized for energy production by intact mitochondria [19]. This may be a common avenue in the pathways involved with neuron protection in PD and ALS.

As previously mentioned, VEGF-A's role in ALS has been extensively studied, compared to VEGF-B, and the mechanism of motor neuron protection by VEGF-A primarily involves VEGFR-2 and Akt/PI3 K activation downstream of VEGFR-2 [54], or suppression of cell death pathways mediated by caspases [55]. With this knowledge, we can get some insight into other pathways involved with VEGFR-1 mediated neuroprotective effects on motor neurons in ALS. There is potential for crosstalk between VEGFR-1 and VEGFR-2 and similar signaling pathways, such as Akt, could be involved. However, knowing that VEGF-A has a prominent effect on vascular permeability compared to VEGF-B and understanding the pathway through which that affect is elicited, then it may be possible to narrow the downstream signals involved in neuroprotection of motor neurons by VEGF-B.

## 8. VEGF-B Action in the Peripheral Nervous System

Studies reveal VEGF-B protection primarily in CNS-derived neurons and actions on the peripheral nervous system have been less characterized. Peripheral nerve injury is major neurological disorder that can cause multiple sensory and motor disturbances. There is a greater capacity of nerves in the peripheral nervous system to regenerate compared to nerves in the central nervous system and successful regeneration requires several factors for axonal regrowth [56]. The family of VEGFs has been associated as a potent mediator of adult nerve regeneration [57]. A study by Guaiquil et al. examined whether VEGF-B mediates peripheral nerve repair after injury from trauma or disease in a mouse model. They revealed restoration of the innervation of target tissues by VEGF-B mediated nerve growth and regeneration and that mice lacking VEGF-B had impaired peripheral nerve regeneration. Additionally, this study showed VEGF-B effects are specific for injured nerves and independent of any vascular effect [34]. Results revealed VEGF-B signaling led to neurite growth in a dose dependent manner and this effect was inhibited by VEGFR-1 antibodies, indicating VEGFR-1 mediated VEGF-B's effects. Additionally, addition of VEGF-B was found to protect against cell death. To further investigate the mechanism by which VEGF-B induces nerve growth, RNA sequencing analysis showed that important elements of the Notch, Wnt, and Plexin signaling pathways may play a role in VEGF-B mediated neurite growth in trigeminal neurons [34]. Previous studies demonstrated that VEGF-A signaling involved PI3 K/Akt [58, 59] so this pathway was also investigated in the context of VEGF-B's mechanism using a specific PI3 K inhibitor. Trigeminal ganglion neurons were pretreated with a PI3 K inhibitor and then incubated with VEGF-B. Neurite growth was followed and the number of neurons showing elongation patterns was analyzed. This analysis revealed that cells treated with the inhibitor formed nascent or short neurites compared to cells treated with VEGF-B alone and elongation of neurites was inhibited as well, proving signaling through PI3 K is required for VEGF-B-induced peripheral neuron growth [34]. Overall, this data demonstrated VEGF-B promotes proper nerve regeneration without affecting undamaged nerves or neovascularization and gave insight into the mechanisms involved, making it an important therapeutic target for treating injured peripheral nerves. In a recent study, VEGF-B improved corneal sensation and epithelial regeneration in both normal and diabetic mice, accompanied with the elevated corneal content of pigment epithelium-derived factor (PEDF). PEDF knockdown partially abolished trophic function of VEGF-B in diabetic corneal reinnervation [60]. PEDF is known to be a master regulator of apoptosis in many tissues and therefore could be driving the antiapoptotic effects of VEGF-B (Figure 1) [61]. This is of particular interest for dopaminergic neurons, as McKay et al. had shown in prior work that PEDF accounts for over 50% of the trophic potential of conditioned media from human retinal pigment epithelial (RPE) cells [62]. And in a follow-up study our group demonstrated that* in vitro* in rat midbrain cultures PEDF protected against both 6-OHDA and rotenone challenges, leading to increased survival and neurite length in dopaminergic neurons [63]. Remarkably, a study that investigated striatal levels of PEDF in PD patients suggests upregulation of PEDF in response to acute insult to the dopaminergic pathway and that such response might be disturbed in patients with advanced PD [64].

## 9. VEGF-B and Cerebral Ischemic Injury

Cerebral ischemia is very common in adults, especially the elderly, and some studies have addressed the potential of VEGF-B to protect against ischemic injury. In this setting, the limited ability of VEGF-B to induce vascular permeability could be advantageous in limiting stroke-related cerebral edema [65].

The effect of cerebral ischemia on other growth factors, such as VEGF-C and PlGF, has been reported. Ischemic injury upregulates VEGF-C in a rat stroke model [66] and PlGF expression was increased in response to oxygen and glucose deprivation in a rat model [67]. The effect on VEGF-B, however, is less clear. To address this, VEGF-B protein expression and protein distribution were measured up to 1-week after middle cerebral artery occlusion in rats. VEGF-B expression was increased in the border zone after injury and was associated with neurons. Therefore, VEGF-B may contribute to adaptive mechanisms that limit ischemic cerebral injury [37].

In a study by Sun et al. the middle cerebral artery was occluded in VEGF-B knockout, heterozygous (HZ), and wild type (WT) mice and the volume of resulting cerebral infarcts in addition to the associated neurological function impairment was measured. To investigate the protective effects of VEGF-B, middle cerebral artery occlusion was produced and infarct areas were measured 24 hours later. Infarct volume was increased approximately 40% in VEGF-B^−/−^ mice compared to VEGF-B^+/+^ mice and confirmed with hematoxylin staining. To assess neurological function, neurological scores were used, which reflect the severity of brain dysfunction after ischemia with higher values indicating more severe impairment. VEGF-B^−/−^ mice received higher scores than did WT and HZ mice 24 hours after middle cerebral artery occlusion. This study concluded the size of cerebral infarcts as well as the severity of neurologic deficits increased in mice lacking the* Vegf-b* gene suggesting that VEGF-B limits the extent of cerebral ischemic injury [37].

The mechanism underlying this neuroprotection is unclear but previous studies as well as the study conducted by Sun et al. provide some insight. VEGFR-1 receptors are involved, as these are the receptors VEGF-B preferentially binds to [68] and it may have a direct action at this site. Comparing VEGF-A and VEGF-B mediated signaling pathways can also provide information on the downstream activity in this context. Protection of neurons from cerebral ischemia by VEGF-A likely involves VEGFR-2 and PI3 K/Akt signaling transduction pathways may be activated [69]. It is possible that VEGF-A and VEGF-B exhibit an additive effect on neurons and protection involves crosstalk between downstream signals of both VEGFR-1 and VEGFR-2.

From another perspective, mitochondrial damage during cerebral ischemia is a possibility due to the effects of increased arachidonic acid levels in the brain. Accumulation of arachidonic acid may inhibit mitochondrial ATP production during cerebral ischemia [70]. This process could be an area in which VEGF-B also plays a role and is a potential area of future investigation. Since VEGF-B expression is upregulated in neurons after ischemic injury [37], VEGF-B might coordinate mitochondrial function via long chain fatty acid uptake, providing an energy source for viable cells. This way, VEGF-B protects against further mitochondrial damage and increases survival of cells during ischemic injury.

## 10. VEGF-B and Cardiac Disease

VEGF-B is expressed in many tissues, including the heart, but the role of VEGF-B in vascular biology is somewhat elusive. It seems VEGF-B is required for normal cardiac function in adult animals. In VEGF-B^−/−^ mice there were no observed gross abnormalities but when assessing cardiac function, ECG showed that VEGF-B^−/−^ mice have an atrioventricular conduction defect, which was characterized by a prolonged QT interval [71]. Despite appearing overtly normal, VEGF-B deficient mice had mild cardiac phenotypes such as smaller heart size and displayed slight vascular dysfunction after coronary occlusion in one strain. Additionally, VEGF-B^−/−^ mice showed clinical symptoms of compromised recovery from induced myocardial ischemia [72]. These results suggest an essential role of VEGF-B in establishing a fully functional cardiovascular system.

Several studies have indicated a role for VEGF-B in cardioprotection. Heart failure is a staggering clinical health problem and is associated with significant mortality and morbidity, particularly among those aged 65 and over, and its prevalence is rapidly increasing [73]. About half of patients with heart failure have diastolic dysfunction [74] but the mechanisms responsible for diastolic heart failure are not well defined.* Vegf-b* gene transfer in rats resulted in prevention of angiotensin II induced left ventricular diastolic dysfunction [75]. Rats were subjected to pressure overload by angiotensin II infusion for 2 weeks, which decreased* E/A* ratio and prolonged left ventricular isovolumic relaxation time. To evaluate the effect of VEGF-B on cardiac function, an echocardiogram was performed, which showed gene transfer ameliorated angiotensin II induced diastolic dysfunction and normalized* E/A* ratio. Additionally, an increased number of cells positive for phosphorylated Akt were induced by VEGF-B gene transfer at 2 weeks [75]. This study demonstrated the association of VEGF-B with induction of the Akt pathway and the important role of VEGF-B in preventing the progression to heart failure. Though there are several drugs that have been reported to influence diastolic function, such as angiotensin-converting enzyme inhibitors and angiotensin receptor blockers, left ventricular hypertrophy occasionally persists and patients are at risk for developing heart failure; thus, VEGF-B serves as a potential therapeutic option.

Normal cardiac development and function as well as repair of damaged and diseased myocardium heavily rely on signaling between cardiomyocytes, endothelial cells, smooth muscle cells, and fibroblasts [76]. Recently, a link was found between VEGF-B and tissue metabolism regulation [77]. A better understanding of factors regulating myocardial angiogenesis and metabolism could lead to the development of new therapies for treatment of heart failure, which, as mentioned, is one of the most common causes of morbidity and mortality in developed countries. Elucidating on the role that VEGF-B plays in this process was the goal of a study conducted by Kivelä et al. in which VEGF-B was shown to increase functional coronary vasculature, reprogram cardiomyocyte metabolic pathways, and protect the rat heart from ischemic damage [40].

An experiment was conducted in which VEGF-B transgenic rats and WT rats were subjected to experimental myocardial infarction by ligating the left coronary artery. Positron emission topography showed a significantly smaller infarct region, 4 weeks after infarction. Furthermore, there was less oxygen consumption in noninfarct transgenic myocardium and transgenic hearts showed a better perfusion of noninfarcted septum as well as a better residual perfusion of the infarcted and border areas. Postmortem histological analysis confirmed the infarct areas and scar tissue areas were smaller in VEGF-B transgenic hearts.

Additional results revealed VEGF-B greatly expands the coronary vasculature and increases functional coronary reserve in VEGF-B transgenic hearts in which there was at least a doubling of number of arteries of all size classes. Lastly, the cardiac hypertrophy seen in VEGF-B transgenic rats is physiologic and the nature of this hypertrophy was determined by analyzing the expression of pathological remodeling genes. There were no differences in gene expression, confirming VEGF-B induced hypertrophy was physiological rather than pathological [40]. The study by Kivelä et al. provided evidence for VEGF-B as a protective and repair-enhancing protein in ischemic heart failure, as its overexpression increases coronary vasculature and reprogramming of myocardial metabolism to improve cardiac function [40].

Mechanisms involved in the protective role of VEGF-B are mediated through several signaling pathways. VEGF-B activated Erk 1/2, Akt, and mammalian target of rapamycin complex 1 (mTORC1) pathway components, indicating VEGF-B signaling engages major regulators of metabolism and cell growth [78] (Figure 1). Activation of mTOR regulates apoptotic pathways that can be dependent on activation of Akt and Erk 1/2. The protective pathways of mTOR can limit cell death to promote cardiac repair and regeneration [40, 79] (Figure 1).

Oxidative stress plays a crucial role in onset of cardiovascular injury and can affect multiple systems that affect metabolic homeostasis. Oxidative stress can be caused by reactive oxygen species and promote cell injury [80]. The mTORC1 pathway has been related to the regulation of cardiac response to stress and myocyte survival [81]. On the other hand, prolonged activation of mTOR can lead to vascular dysfunction [82]; therefore, a balance is crucial to promote favorable effects.

Cardiac fatty acid uptake was also analyzed since it was reported that VEGF-B upregulates endothelial fatty acid transport via FATP3 and FATP4 [42]. Though results from Kivelä et al. confirm VEGF-B has metabolic effects in the heart, it does not occur at the substrate level. Rather, VEGF-B mediates metabolic signaling pathways via 5′ AMP-activated protein kinase (AMPK) and mTORC1 and directs fatty acids to synthetic pathways rather than oxidation of fatty acids [40].

This information provided a better understanding of the signals involved in VEGF-B mediated protection of cardiomyocytes from cardiac injury and can be used to hypothesize the role of VEGF-B in pathways involving mTOR in neurodegenerative disease, specifically PD. In PD, mTOR activation can prevent injury of dopaminergic neurons during oxidative stress [83, 84]. Given that mTOR is a downstream target of the Akt and Erk 1/2 pathways as previously mentioned, and VEGF-B has been shown to upregulate these pathways in a PD model system [19], it is possible that VEGF-B's protective effects involve mTOR downstream of increased Akt and Erk, reducing apoptosis and protecting neurons from injury (Figure 1).

Overexpression of VEGF-B in the heart also leads to increased fatty acid synthesis via downregulation of phosphorylated 5′ AMP-activated protein kinase (pAMPK) and an increase in Malonyl-CoA and fatty acid synthase (FASN) downstream. The increase in fatty acid synthesis provides fatty acids for mitochondrial use and energy production and serves as another mechanism of cardiomyocyte survival [85]. Again, this is another potential area of future research for VEGF-B's mechanism of action in neuroprotection (Figure 1) identified using information about VEGF-B's action in the cardiac system. A caveat for this mechanism contributing to ATP generation is that in neurons this could constitute a “futile” cycle in terms of energy production, as the concomitant increase of Malonyl-CoA will drain the mitochondria of citric acid, whereby more NADH but no additional ATP might be generated. On the other hand, AMPK could be involved in dopaminergic neurons in a separate way, as Kang et al. demonstrated that *α*-synuclein binds phosphoinositide-3 kinase enhancer L in a phosphorylation dependent manner and sequesters it in Lewy bodies, leading to dopaminergic cell death via AMPK hyperactivation [86]. Given the role of Lewy bodies and abnormal aggregation of *α*-synuclein in the pathogenesis of PD, reduction of pAMPK could be an alternative way in which VEGF-B could be protective for dopaminergic neurons.

## 11. VEGF-B and PGC-1*α*

These effects of VEGF-B as a homeostatic metabolic regulator are complex and much remains to be learned about the specific role of VEGF-B in metabolic regulation. To elucidate on this role, data from a study looking into peroxisome proliferator-activated receptor gamma coactivator 1-*α* (PGC-1*α*) in skeletal muscle was taken into consideration, where VEGF-B was shown to be a downstream target of the PGC-1*α* signaling pathway [87]. Additionally, in a study by Huusko et al., AAV9-VEGF-B gene transfer was able to postpone the development of heart failure, and to evaluate the degree of metabolic remodeling, the mRNA levels of PGC-1*α* were measured. Interestingly, after the AAV9-VEGF-B gene therapy, mRNA expression of PGC-1*α* was significantly increased [88]. This is suggestive of a positive feedback loop in which VEGF-B is directly upregulating PGC-1*α* followed by increased mitochondrial activity, or VEGF-B could be increasing mitochondrial activity that is then feeding back to increase PGC-1 *α* in order to maintain increased production of ATP (Figure 1). Since PGC-1*α* can upregulate NRF1 and NRF2* (vide infra)*, it is possible that these transcription factors are involved in this direct positive feedback regulation in the center of VEGF-B-induced neuroprotection (Figure 1).

Given that PGC-1*α* is a central regulator within mitochondrial function that provides a protective effect in both cardiomyocytes and neurons, we propose that VEGF-B may tie into these pathways in several ways. In cardiac cells, transcription factors NRF1 and NRF2 are activated by PGC-1*α*, contributing to mitochondrial membrane biogenesis [89]. In neuroprotection, PGC-1*α* also increases activity of these transcription factors [90]. In postmortem brains of PD patients, in both the SNpc and blood cells, PGC-1*α* levels are decreased [91] and in PGC-1*α* knockout mice, dopaminergic cells are more sensitive to MPTP [92]. Another study demonstrated PGC-1*α* overexpression reduced *α*-synuclein levels, in addition to protecting cells from toxic effects of *α*-synuclein [93]. Overall, these studies reveal PGC-1*α*'s neuroprotective role.

In both systems, it is possible that VEGF-B not only is a downstream target of PGC-1*α*, but also is linked with it in a feedback loop. In the context of neuroprotection of dopaminergic neurons, PGC-1*α* reduces ROS and provides positive effects on mitochondrial function. VEGF-B may play a role in this PGC-1*α* regulated event by increasing long chain fatty acid uptake via FATP1 and FATP4, improving mitochondrial function and reducing oxidative injury (Figure 1). This could be further enhanced if the increase of fatty acid syntheses, described for cardiac cells above, also happens in dopaminergic neurons. A recent small pilot study from our group does indicate that long-term viral overexpression of VEGF-B [94] might lead to increased striatal dopamine content in PINK1-knockout rats [95], a rodent model for a familial form of PD thought to involve mitochondrial defects. This could indicate long-term neuroprotective activity of VEGF-B for dopaminergic neurons with impaired mitochondrial function and needs further study.

## 12. VEGF-B and the Inverse Warburg Effect

There is an intriguing recent theoretical framework that could explain the protective action of VEGF-B on a systems level, involving the inverse Warburg effect [96]. A proposed model by Warburg postulates that cancer is a metabolic disease induced by abnormalities in mitochondria. The subsequent metabolic alteration in this model is the upregulation of glycolysis to compensate for diminished energy. This reprogramming is known as the Warburg effect and is derived from the observation that most cancer cells can switch to a less efficient form of metabolism and produce energy necessary for proliferation via glycolysis [97]. The concept of the inverse Warburg effect was introduced by Demetrius and colleagues to describe a complementary mode of metabolic reprogramming occurring in Alzheimer's disease [96, 98]. It has since been hypothesized to also be a contributing factor to the mitochondrial dysfunction seen in dopaminergic neurons that leads to PD, since a hypermetabolic state, as predicted by the inverse Warburg effect, has been detected in the brains of individuals during the presymptomatic phase of PD [99]. This effect initiates a neurodegenerative cascade, which involves a disproportionate upregulation of oxidative phosphorylation in mitochondria of certain neurons to compensate for energy production impairment. Cells not only are dependent on carbohydrate-derived pyruvate but also become critically dependent on lactate-derived pyruvate, with the lactate provided by astrocytes, ultimately leading to a competition for lactate among normal and impaired neurons. This resource constraint allows impaired neurons with upregulated oxidative phosphorylation activity to outcompete intact neurons with normal oxidative phosphorylation activity. Subsequently, there is a change in the overall neuronal population distribution, with an increase in the proportion of impaired neurons compared to healthy neurons [98]. While the inverse Warburg effect is still quite hypothetical in PD, particularly because there is no evidence for an upregulation of complexes of the respiratory chain in dopaminergic neurons in PD, it does provide an intriguing novel theoretic framework to help explain protective effects mediated via enhanced fatty acid metabolism. Providing alternative fuel sources, such as fatty acids, may reduce this resource constraint and alleviate the advantage of impaired neurons. The homeostatic regulator VEGF-B, in a feedback loop together with PGC-1*α*, could increase mitochondrial function via fatty acid uptake and usage, such that lactate is no longer a limiting resource for the neurons. This process could help prevent deleterious effects on initially healthy neurons and potentially serve to suppress the inverse Warburg effect, reducing the neurodegeneration of the starved neurons.

## 13. Conclusion

The aim of this review was to elucidate the mechanism of VEGF-B action in different systems and by taking data from both heart and skeletal muscle, we can infer possible functions and mechanisms of VEGF-B in neurons, particularly in the case of dopaminergic neurons in PD. Despite the complex etiology of neurodegenerative diseases, a common theme among them is mitochondrial dysfunction and apoptotic neuronal death. Numerous studies demonstrate VEGF-B is a potent protective factor with a unique safety profile. Ultimately, VEGF-B seems to possess a greater safety advantage, given the lack of angiogenic function, which is an important consideration for its potential therapeutic use in human diseases. There is evidence that VEGF-B promotes energy metabolism and a common observation between the pathogenesis of the diseases discussed in this review is the energy dependent nature of the cells involved. VEGF-B is a factor involved in the crosstalk between oxidative metabolism and mitochondrial biogenesis. There is strong evidence suggesting VEGF-B plays a crucial protective role, whether in context of heart failure or neurodegeneration. Though mechanistically there are differences in VEGF-B action in different tissues, there are also similarities in the downstream regulators involved in VEGF-B's effect. These similarities are a point of convergence, which strongly supports the importance of further illuminating the role of VEGF-B and its use as a therapeutic agent during neuronal injury. Therefore, it would be worth investigating VEGF-B's mechanisms of action further in dopaminergic neurons in the future to ultimately develop a VEGF-B targeted therapy that results in improving mitochondrial dysfunction in the context of cardiac disease and neurodegenerative disorders, including Parkinson's disease.
